# Extracellular Vesicles in the Mesenchymal Stem Cell/Macrophage Axis: Potential Targets for Inflammatory Treatment

**DOI:** 10.3390/ijms26062827

**Published:** 2025-03-20

**Authors:** Zhen Che, Wenbin Yan, Qun Zhao

**Affiliations:** 1 Experimental Orthopedics and Trauma Surgery, University Hospital RWTH Aachen, Pauwelsstraße 30, 52074 Aachen, Germany; zche@ukaachen.de; 2 Department of Orthopedics, Sun Yat-sen Memorial Hospital, Sun Yat-sen University (SYSU), Guangzhou 510120, China

**Keywords:** extracellular vesicles (EVs), mesenchymal stem cells (MSCs), macrophages (Mφ), mesenchymal stem cell/macrophages axis (MSC/Mφ axis), inflammatory diseases, MicroRNAs (miRNAs)

## Abstract

Mesenchymal stem cells (MSCs) have been widely used for the treatment of autoimmune and inflammatory diseases due to their pluripotent differentiation potential and immunomodulatory function. Macrophage (Mφ) polarization also acts an essential and central role in regulating inflammation, basically the dynamic balance of pro-inflammatory M1-like (M1φ) and anti-inflammatory M2-like macrophages (M2φ), affecting the occurrence and progression of inflammatory diseases. Since a pivotal molecular crosstalk between MSCs and Mφ has been elucidated using in vitro and in vivo preclinical studies, we presume that the mesenchymal stem cell/macrophages axis (MSC/Mφ axis) acts an important role in pathophysiological mechanisms of inflammatory diseases and should be the potential therapeutic target. However, the crucial effects of EVs as intercellular communicators and therapeutic agents in the MSC/Mφ axis remains explorable. Therefore, this review elaborated on the mechanisms of EVs mediating the MSC/Mφ axis regulating inflammation in-depth, hoping to provide more references for related research in the future.

## 1. Introduction

Inflammatory diseases, especially chronic non-infectious inflammatory diseases, including atherosclerosis [1], rheumatoid arthritis (RA) [2] and inflammatory bowel disease (IBD) [3] among others, have caused great social–economic burdens on patients. Although there various treatments have been proposed to inhibit inflammatory responses and protect tissues from irreversible destruction, few target the underlying mechanisms. Thus, although inflammation is temporarily under control, the termination of current therapies or reduction in dosage can exacerbate original inflammatory responses and aggravate patients’ condition [2]. Therefore, it is significant that the mechanisms of diverse inflammatory diseases can be elucidated in-depth, as well as that state-of-the-art therapies can be investigated accordingly.

Macrophage (Mφ) polarization plays an essential and central role in regulating inflammation, referring to the dynamic balance of M1-like (M1φ) and M2-like macrophages (M2φ), affecting the occurrence and progression of inflammatory diseases [4]. Briefly, M1φ are considered as the pro-inflammatory phenotype which promote the progression of IBD, acute lung injury (ALI), mastitis and idiopathic pulmonary fibrosis, while the anti-inflammatory type M2φ can inhibit inflammatory responses and alleviate asthma, liver injury, myocardial infarction and so on [5]. Hence, the modulation of Mφ polarization could be a potential target for comprehensive treatment and convincing method to understand inflammatory disease pathogenesis.

Mesenchymal stem cells (MSCs) have attracted the attention of researchers from all over the world due to their pluripotent differentiation potential and immunomodulatory function, and they have been widely used in clinical trials for the treatment of autoimmune and inflammatory diseases [6]. However, although therapies based on MSCs are novel approaches for intractable and severe inflammatory diseases, the further expansion of their utilization is constrained by rejection concerns, challenges in accurate differentiation, potential tumorigenicity, heterogeneity, low survival rate and ethical considerations [7,8]. Recently, increasing studies have demonstrated that MSCs’ key role in regulating innate and adaptive immune responses mainly relies on paracrine pathways to exert, indicating that non-cell therapy could be a reasonable solution [9].

Extracellular vesicles (EVs) are currently widely studied as messengers in intercellular communication [10], as well as potential therapeutics for inflammatory diseases due to their properties as natural delivery vehicles for cargoes, such as nucleic acids, proteins, lipids and so on, and high plasticity to be engineered as promising nanoparticles to develop personalized therapeutic carriers [11]. Numerous studies have suggested that mesenchymal stem cell-derived EVs (MSC-EVs) are important parasecretions from MSCs and perform similar therapeutic roles, with great advantages compared to stem cell therapies, such as self-replication inability, lower risk of immune rejection and ectopic differentiation, and both have great genetic stability and perform tumor formation [12]. Meanwhile, macrophage-derived EVs (Mφ-EVs) have been proven to function as a double-edged sword, like their parent cells in inflammation, as vital mediators both in the pathology of inflammatory diseases and of beneficial effects in immunoregulation and tissue repair [13]. More importantly, a pivotal molecular crosstalk between MSCs and Mφ has been elucidated using in vitro and in vivo preclinical studies [14]. Therefore, we consider that the mesenchymal stem cell/macrophages axis (MSC/Mφ axis) acts an important role in pathophysiological mechanisms of inflammatory diseases and should be potential therapeutic target. However, the crucial effects of EVs as intercellular communicators and therapeutic agents in the MSC/Mφ axis remains yet to be explored.

In the current paper, we review the basis of the interaction between MSCs and Mφ, as well as the biological characteristics and functions of EVs. Subsequently, we elaborate on the mechanisms of EVs mediating the MSC/Mφ axis regulating inflammation in-depth. Advanced experimental and clinical studies of EVs in inflammatory diseases are also introduced with their future challenges and prospects. Through the above summary and conclusion, we hope to answer the three following core scientific questions: how do EVs mediate the regulation of MSCs on Mφ polarization? How do molecules carried by EVs affect the inflammatory microenvironment? And finally, what is the feasibility of EVs as therapeutic targets or drugs?

## 2. Basis of the Interaction Between MSCs and Mφ

### 2.1. Dual Roles of Mφ

Mφ play dual roles in the inflammatory process due to their two main phenotypes, M1φ and M2φ. M1φ, the pro-inflammatory type Mφ, has excessive activities that are always accompanied with the high-level expression of M1-related genes, such as interferon regulatory factor 5 (IRF5), signal transducer and activator of transcription 1 (STAT1) and inducible nitric oxide synthase (iNOS), cytokines including tumor necrosis factor α (TNF-α) and interleukin 6 (IL6), and Cluster of Differentiation 86 (CD86) [15]. In comparison, the expression of M2-related genes including IRF4, STAT6 and Arg1, as well as cytokines such as IL10 and CD206, are promoted, while the anti-inflammatory M2φ is induced [15]. The dynamic balance of M1φ and M2φ is quite important in the occurrence, progression and prognosis of inflammatory diseases. Moreover, it was found that Mφ-EVs and their contents also have significant functions in the pathophysiological mechanisms of a variety of inflammatory diseases, indicating great potential for being biomarkers, therapeutics and drug delivery vehicles [16].

More specifically, the imbalance of Mφ homeostasis is considered as a dominating contributor which promoting inflammation in chronic non-infectious inflammatory diseases, like RA and pulmonary fibrosis, namely less and inactive M2φ and more active M1φ [17]. Therefore, modulating the Mφ polarization becomes the primary goal for treatment targeting underlying mechanisms. While glucocorticoids or other disease-modifying antirheumatic drugs (DMARDs) can induce the repolarization of M1φ toward M2φ, scientists have been committed to studying more precise drug delivery or treatment methods with fewer side effects [17]. Naturally, EVs with homing capability entered researchers’ sights and their flexibility to be engineered has attracted more attention.

### 2.2. Biological Characteristics of MSCs

MSCs are characterized by multi-potent differentiation, low immunogenicity, immunomodulation capacity and other biological characteristics [18]. They can originate from the umbilical cord (UC-MSCs) [19], amniotic (AMSCs) [20], bone marrow (BM-MSCs) [21] and adipose tissue (AD-MSCs) [22], among others. In addition, under appropriate culture environment or stimulation, MSCs can be induced to become a variety of different cells and play corresponding functions. Therefore, MSCs have gradually become the most used cells in stem cell therapies [23]. In recent years, many researchers have worked on demonstrating the mechanisms of MSCs’ immunomodulation capacity. Their results suggested that through secreting various substances via paracrine pathways, for example, EVs and MSCs show positive effects in multiple inflammatory diseases [24]. As the study deepens, increasing numbers of scientific researchers consider that parasecretions of MSCs, especially EVs, play vital roles in the cellular communication and immunomodulation capacity of MSCs, and are able to exert similar functions independently [25].

MSCs act as the “sensor” and “modulator” in inflammatory microenvironment. As the “sensor”, MSCs can actively sense the dynamic changes in the inflammatory microenvironment like hypoxia and mechanical stress, recognize inflammatory signals through multiple molecular mechanisms and initiate adaptive responses. For instance, Toll-like receptors (TLRs) [26] expressed on the surface of MSCs can recognize pathogen-associated molecular patterns (PAMPs) [27], such as bacterial lipopolysaccharide (LPS), or damage-associated molecular patterns (DAMPs), such as high-mobility group box 1 protein (HMGB1) [28], and then trigger downstream signaling pathways, such as nuclear factor kappa-light-chain-enhancer of activated B cells (NF-κB) [28,29]. On the other hand, as the “modulator”, MSCs actively regulate immune cells (such as Mφ, T cells) and inflammatory processes in the inflammatory microenvironment by secreting soluble factors, cell-to-cell contact, or releasing EVs [30]. For example, MSCs can exert their immunosuppressive properties through educating B cells and inducing regulatory B cells (Bregs) proliferation [31]. The collaboration between the roles of the “sensor” and “modulator” of MSCs making them ideal therapeutic tools for inflammatory diseases; however, it is still necessary to analyze its dynamic regulatory mechanism in-depth to avoid potential risks.

### 2.3. Classical Mechanisms of MSCs Regulating Mφ Polarization

Through various intercellular communicational mechanisms, MSCs regulate the Mφ polarization in immune-mediated inflammatory diseases (IMIDs), such as cell–cell contact, efferocytosis, soluble molecules, mitochondria transmission and so on. For example, MSCs can secrete cytokines, such as IL10 and TGF-β, which directly inhibit M1φ polarization and promote M2φ phenotype. In addition, through surface molecule binding, like CD200-to-CD200 receptors (CD200R), MSCs can transmit inhibitory signals to macrophages as well. Moreover, Mφ glycolysis-mediated inflammatory response can be inhibited via inhibiting hypoxia-inducible factor 1α (HIF-1α) expression to reduce M1φ polarization, namely metabolic reprogramming pathways [24]. There are also several classical signaling pathways involved in mechanisms of MSCs regulating Mφ polarization, including STAT, NF-κB and Notch signaling pathways. Microenvironment regulation, like hypoxic conditions and efferocytosis, also play important roles in MSC/Mφ axis [32].

Recently, EVs have been proposed as new regulatory vehicles in the MSC/Mφ axis. It was suggested that MSC-EVs or their contents can be internalized into Mφ through endocytosis, or receptor molecules on the surface of Mφ can receive signals from contents like micro-RNAs (miRNAs) and proteins, including miR-223 targeting PBX/Knotted 1 Homeobox 1 (PKNOX1), miR-181c inhibiting TLR4/NF-κB signaling pathways and miR-21 promoting M2φ through phosphatase and tensin homolog/protein kinase B (PTEN/Akt)-signaling pathways, producing various biological effects, including the M2φ polarization-promoting effect [33].

## 3. Biological Characteristics and Functions of EVs

### 3.1. Classification and Biogenesis of EVs

**Figure 1:**
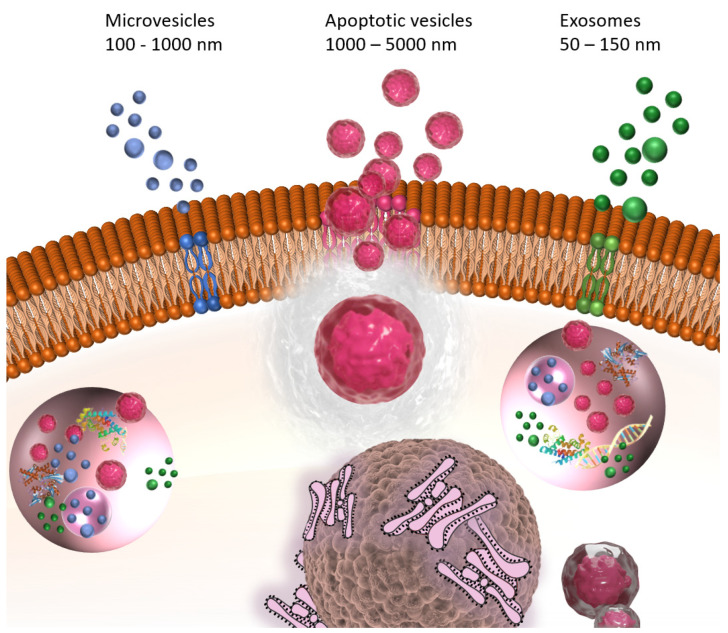
Formation and release of exosomes, microvesicles (MVs) and apoptotic vesicles (ApoVs).

EVs, including exosomes, microvesicles (MVs), and apoptotic vesicles (ApoVs), as shown in Figure 1, are recognized as promising therapeutic vehicles due to their intrinsic biocompatibility and nano-size, allowing them to penetrate inner physiological barriers, like the blood–brain barrier (BBB) [34,35]. Exosomes are characterized by the smallest EVs, with their sizes ranging from 30 to 150 nm. All cell types, even including synovial fluid, can secrete exosomes. However, their obtainment could be difficult because of their heterogeneity, contamination and the complexity of biological fluids [36]. MVs, another subset of large-sized EVs, originate and shed from plasma membrane [37]. ApoVs, once only regarded as apoptotic cell cleaners, are produced during the apoptosis process, with various biological activities [38]. Although they vary in size and bio-occurrence, due to their similar composition and structure, containing nucleic acids like miRNA and long non-coding RNA (lncRNA), proteins such as cytokines and membrane receptors, lipids, etc., which come from their parent cells, have common functionalities that are worth studying as natural molecular delivery vehicles with high plasticity.

MSC-EVs are membrane structures secreted by MSCs that are rich in specific proteins, lipids and nucleic acids. Among them, miRNAs have received the most attention so far. Unmodified MSC-EVs can promote or inhibit tumor growth, while modified MSC-EVs participate in inhibiting cancer progression by delivering therapeutic molecules (including miRNA, specific siRNA or suicide RNA), as well as chemotherapeutic drugs [39]. In addition, there are also differences between EVs from different types of MSCs, including UC-MSCs, AMSCs, BM-MSCs and AD-MSCs [40].

### 3.2. Intercellular Communication Mechanisms of EVs

EVs mediate intercellular communication through multiple mechanisms which can be divided into two categories. Firstly, targeted delivery can be conducted by combination of integrins or tetraspanins (CD9/CD81) on the EVs membrane with receptor cell surface ligands, such as intercellular adhesion molecule-1 (ICAM-1) [41]. Besides surface molecules, EVs carrying chemokine receptors such as CXCR4 can also be attracted to specific microenvironments. Secondly, content transfer between EVs and target cells via membrane fusion, endocytosis and signal transduction is another primary mechanism. EVs can directly fuse with the receptor cell membrane and release their contents into the cytoplasm (e.g., synaptic transmission between neurons). In addition, recipient cells can take up EVs through clathrin-mediated endocytosis, macropinocytosis or phagocytosis [42]. Moreover, EVs surface molecules such as the Fas ligand (FasL) and major histocompatibility complex (MHC) peptide complexes, can directly activate receptor cell membrane receptors, including death receptors or T cell receptors.

## 4. Mechanisms of EVs Mediating the MSC/Mφ Axis Regulating Inflammation

### 4.1. Key Molecules in Contents of EVs

As derivatives, EVs carry a series of key molecules from their parent cells like MSCs or Mφ and modulate inflammatory responses via multiple pathways. miRNAs are one of the most important bioactive molecules carried, and function from upstream to downstream on the MSC/Mφ axis in inflammation regulation. According to an experimental research, scientists applied engineered EVs to suppress peripheral immune cells [37]. The results showed that the engineered EVs performed excellent capacity to promote regulatory T cells (Tregs) induction and anti-inflammatory M2φ polarization through the upregulation of miR-155-3p. Another interesting study indicated that EVs derived from various normal tissues, which can be differentiated from MSCs, could coordinate Mφ homeostasis and mitigate inflammatory damage [43]. EVs-treated Mφ exhibited LPS resistance, reduced expression of inflammatory cytokines and enhanced phagocytic activity. In addition, miRNAs, including miR-148a-3p, miR-1a-3p and miR-143-3p, were found abundant in EVs, promoting the resolution of LPS-induced inflammation in Mφ by multiple pathways, such as STAT3, NF-κB p65 and c-Jun N-terminal kinase (SAPK/JNK).

Downstream of the MSC/Mφ axis, an animal experiment suggested that miR-709 in M2-like macrophages-derived EVs (M2φ-EVs) could partially mediate protective effects of M2φ-EVs for acute lung injury/acute respiratory distress syndrome (ALI/ARDS) [44]. In the LPS-induced ALI mouse model, decreasing endogenous M2φ-EVs were found, and exogenous ones could inhibit the pyroptosis of Mφ and the excessive release of cytokines such as IL6, TNF-α and IL-1β, both in vivo and in vitro. Mechanistically, the inhibition of the NF-κB/NLR family pyrin domain-containing 3 (NLRP3) signaling pathway was closely related, and the expression of miR-709 was positively correlated with the protective effects of M2φ-EVs [44]. On the contrary, in periodontitis (PD) caused by porphyromonas gingivalis (Pg), ApoVs derived from Mφ were found enriched with miR-143-3p, targeting insulin-like growth factor-binding protein 5 (IGFBP5), thereby disrupting periodontal bone homeostasis [45]. Additionally, researchers proved that during inflammation, miR-92a-3p upregulation could be induced via the TLR4/miR-92a-3p/PTEN/NF-κB unidirectional pathways, increasing the production and secretion of sclerostin (SOST) from RAW 264.7 cells, a type of Mφ, in the form of EVs. Along with many other articles, miRNAs of EVs were proved to have potential as key targets and biomarkers on the MSC/Mφ axis such as miR-625-3p, and miR-671-5p from MSC-EVs targeting adaptor-associated protein kinase 1 (AAK1) in inflammatory lung diseases [46,47,48].

Although some researchers proposed that proteins contained by EVs are more relative with the activation of inflammation, while miRNAs restrict the over-action of Mφ in inflammatory stimulation, there are still numerous anti-inflammatory proteins carried by EVs function positively on the MSC/Mφ axis [43]. Tumor necrosis factor-inducible gene 6 protein (TSG6) inhibits the activation of the TLR2/NF-κB signaling pathway by binding to the Mφ surface receptor CD44, reducing the release of pro-inflammatory factors such as TNF-α and IL6, thereby inhibiting the inflammatory cascade reaction [49]. Moreover, TSG-6 promotes the formation of an anti-inflammatory microenvironment by inhibiting an M1φ polarization marker, such as iNOS and IL-1β, and upregulating M2φ markers, including Arg1 and CD206. In addition, TSG6 can also cooperate with the STAT signaling pathway to enhance its anti-inflammatory effect. A study showed that when MSCs were co-cultured with Mφ, pro-inflammatory factors, such as TNF-α, IL-1β, and interferon gamma (IFN-γ), significantly upregulated TSG-6 expression by activating the Janus kinase (JAK)/STAT 1/3 pathway [50].

Prostaglandin E_2_ (PGE_2_) has also been proven as an anti-inflammatory protein on the MSC/Mφ axis. Under the stimulation of inflammatory factors like TNF-α and IL-1β, MSCs upregulate the expression of cyclooxygenase-2 (COX-2) and catalyze arachidonic acid to produce PGE_2_. PGE_2_ activates the cyclic adenosine monophosphate/protein kinase A (cAMP/PKA) pathway by binding to the PGE_2_ receptor 2/4 (EP2/EP4) on the surface of Mφ, inhibits NF-κB activity and promotes the secretion of anti-inflammatory factors such as IL10, inducing the transformation of Mφ to the Mφ2 phenotype [51]. In addition, PGE_2_ can inhibit the synthesis of pro-inflammatory factors, such as IL12 and TNF-α in Mφ, blocking the activation of NLRP3 inflammasomes and reducing the maturation and release of IL-1β [44,52]. Meanwhile, it is believed that in the inflammatory microenvironment, TSG-6 and PGE_2_ amplify the anti-inflammatory effect through synergistic action.

Metabolites such as lactate can shape the microenvironment and modulate Mφ metabolic reprogramming to promote M2φ polarization. EVs can transfer lactate or lactate modification-related enzymes, such as lactate dehydrogenase (LDHA), promoting the accumulation of lactate in macrophages [53]. Lactylation can inhibit the expression of pro-inflammatory genes (iNOS, etc.) and activate repair-related genes (Arg1, etc.), promoting the M2 polarization. It was also reported that the Warburg effect can be inhibited by lactate through activating pyruvate kinase M2 (PKM2) to inhibit glycolysis and reduce lactate production, thereby reversing the metabolic adaptation of pro-inflammatory Mφ, promoting the transition of M1φ towards a reparative phenotype [54]. Acidic microenvironments also have synergistic effects. Lactate released by EVs lowers the extracellular pH, forming an acidic microenvironment, which further inhibits T cell activity and enhances the immunosuppressive function of Mφ [55].

### 4.2. Signaling Pathway Regulation

**Figure 2:**
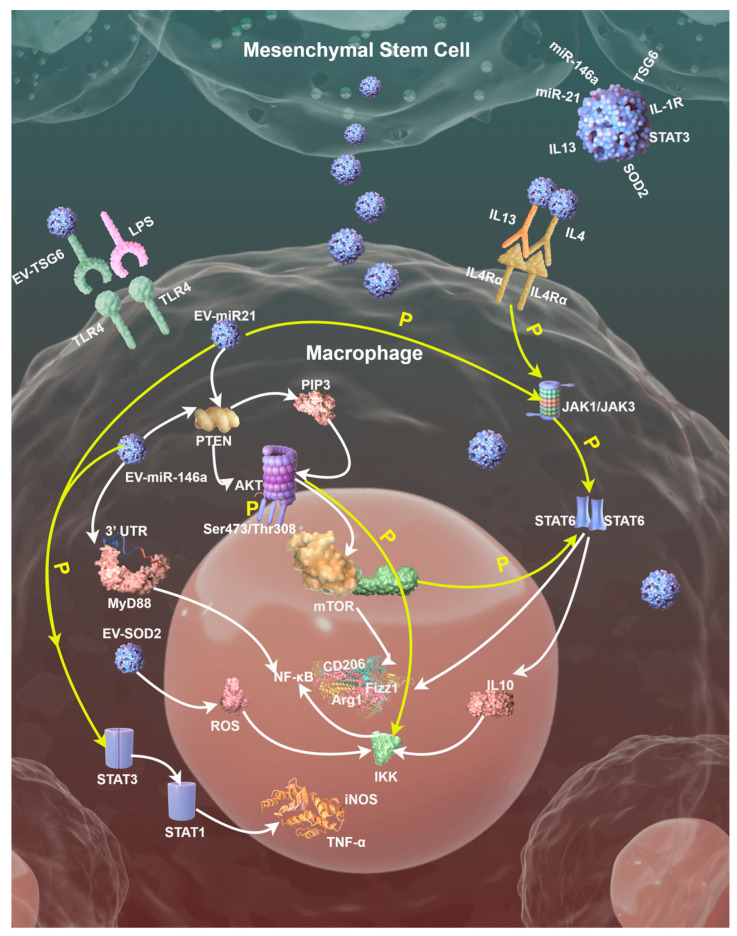
A schematic diagram displaying three signaling pathways involved in modulation mechanisms of EVs on the MSCs/Mφ axis; tiny blue balls represent different EVs. Diagram drawn by Figdraw, an open graphics platform (https://www.figdraw.com/static/index.html#/, accessed on 21 February 2025).

In this section, we introduce three signaling pathways involved in modulation mechanisms of EVs on the MSCs/Mφ axis, which are shown in Figure 2.

#### 4.2.1. TLR/MyD88/NF-κB Signaling Pathway

EVs effectively inhibit pro-inflammatory responses by intervening in the TLR/Myeloid differentiation primary response 88 (MyD88)/NF-κB signaling pathway through multiple targets. Mechanistically, its action involves the regulation of the entire chain from receptor activation to the expression of downstream inflammatory factors. Firstly, EVs can block the ligand recognition and activation of TLR4. MSC-EVs contain TLR4 antagonists like TSG-6, which reduce TLR4 dimerization and downstream signaling initiation by binding to TLR4 or competitively inhibiting the recognition of its ligands, such as LPS [56]. EVs membrane components including cholesterol or sphingomyelin can also change the fluidity of Mφ membranes, modulating the membrane microenvironment, then inhibit the interaction between TLR4 and auxiliary proteins, reducing receptor sensitivity.

Secondly, as the core adaptor protein in the TLR signaling pathway, MyD88’s signal transduction can also be regulated by EVs. MSC-EVs can deliver miRNAs such as miR-146a, which directly target the 3′ untranslated region (UTR) of MyD88 messenger RNA (mRNA), inhibit its translation and reduce MyD88 protein synthesis [57]. Meanwhile, signal regulatory proteins such as STAT3 or interleukin-1 receptor (IL-1R)-associated kinase (IRAK)-M in EVs can interfere with the interaction between MyD88 and IRAK family kinases, block the assembly of Myddosome (MyD88-IRAK signalosome) and thus inhibit downstream signal transduction. In a recent study, scientists applied engineered EVs with optimized homing capacity and loaded them with MyD88, successfully delivering therapeutic peptides in inflammatory diseases associated with TLR activation [41].

Thirdly, EVs block NF-κB activation via dual mechanisms, stabilizing the nuclear factor of kappa light polypeptide gene enhancer in B-cells inhibitor alpha (IκBα) protein and regulating NF-κB nuclear localization. Antioxidant enzymes, such as superoxide dismutase 2 (SOD2), carried by EVs reduce the accumulation of reactive oxygen species (ROS), inhibit the phosphorylation of IκB kinase (IKK) complex, prevent the degradation of IκBα, and maintain the inactive state of NF-κB in the cytoplasm [57]. Moreover, miR-21 in EVs activates the Akt pathway by targeting PTEN, enhancing the nuclear retention of NF-κB inhibitors such as p50 homodimers and inhibiting pro-inflammatory gene transcription [56].

#### 4.2.2. PI3K/Akt/mTOR Signaling Pathway

Specific miRNAs carried by EVs like miR-21 and miR-146a can target and inhibit PTEN, thereby relieving its negative regulatory effect on the phosphatidylinositol 3-kinase (PI3K)/Akt pathway. PTEN blocks Akt activation by dephosphorylating phosphatidylinositol-3,4,5-triphosphate (PIP3), while miRNA delivered by EVs reduces PTEN expression, leading to PIP3 accumulation, which in turn activates Akt phosphorylation (Ser473/Thr308 sites). Activated Akt promotes the expression of M2 markers, such as Arg1 and CD206, through the downstream effector molecule, mammalian target of rapamycin (mTOR) [58].

Activated Akt relieves the inhibition of mTOR complex 1 (mTORC1) by phosphorylating the tuberous sclerosis complex 1/2 (TSC1/2), promoting glycolysis and fatty acid oxidation (FAO), and providing metabolic support for M2φ. mTORC1 upregulates HIF-1α, induces LDHA expression and accelerates lactate production [59]. Lactate is fed back to Mφ through EVs, further activating HIF-1α and forming a metabolic cycle that promotes M2φ. mTORC1 can also promote mitochondrial biogenesis through peroxisome proliferator-activated receptor gamma coactivator 1-alpha (PGC-1α) and enhance oxidative phosphorylation (OXPHOS), providing energy for the long-term survival and tissue repair function of M2φ [60].

The synergistic effects between signaling pathways should also not be ignored. Akt can stabilize IκBα protein by phosphorylating IKK, preventing NF-κB nuclear translocation and reducing the release of M1φ markers such as TNF-α and IL6. Meanwhile, Akt-activated mTORC1 enhances STAT6 phosphorylation and promotes the transcription of IL4/IL13-induced M2φ-related genes, including Arg1 and Fizz1 [61].

#### 4.2.3. STAT Signaling Pathway

As described above, EVs can carry IL4 or IL13-like molecules, which activate JAK1/JAK3 kinases by binding to Mφ surface receptors (e.g., IL-4Rα) and then phosphorylate STAT6. Activated STAT6 forms dimers and translocates into the nucleus, directly binding to the promoter regions of M2φ-related genes (Arg1, Fizz1, CD206, etc.) driving their transcriptional expression. In addition, EVs can deliver miR-21 or miR-146a, inhibiting the expression of suppressor of cytokine signaling (SOCS) family proteins SOCS1/3, relieving their negative regulation on the JAK/STAT pathway, thereby enhancing the phosphorylation and activity of STAT3/STAT6 [62,63].

EVs promote STAT3 phosphorylation by delivering metabolites (e.g., α-ketoglutarate) or regulating glutamine metabolism. For example, the SENP1-Sirt3 signaling axis promotes M2φ polarization by enhancing the production of α-ketoglutarate, inhibiting HIF-1α activity and stabilizing STAT3 activation [64]. EVs also inhibit STAT1 phosphorylation by activating STAT3, thereby blocking IFN-γ-induced M1φ polarization. In IL4-pretreated Mφ, STAT3 activation can inhibit the expression of STAT1-dependent pro-inflammatory genes (e.g., iNOS and TNF-α), forming an anti-inflammatory microenvironment.

EVs-activated STAT6 can induce peroxisome proliferator-activated receptor γ (PPARγ) expression, which further consolidates M2φ by lipid metabolism reprogramming, like enhancing FAO and inhibiting NF-κB-mediated pro-inflammatory responses [64]. Furthermore, it upregulates the anti-inflammatory factor IL10, inhibits the activity of the IKK complex and reduces the degradation of IκBα, thereby blocking the nuclear translocation of NF-κB and its mediated release of pro-inflammatory factors.

### 4.3. Microenvironment Remodeling Effect

While LPS-induced inflammation can activate the NLRP3 inflammasome through the TLR/MyD88/NF-κB signaling pathway, promoting inflammatory cascade reaction and aggravating tissue injury, EVs-carried miR-223 targets NLRP3 mRNA, reduces the efficiency of inflammasome assembly and reduces the mature release of IL-1β and IL18 by inhibiting Caspase-1 activation [65]. Through function of EVs on the MSC/Mφ axis, multiple tissue repair factors’ secretion can also be promoted via various mechanisms mentioned above, like Arg-1, IL10 and so on. Also, when acidic microenvironments enhance the immunosuppressive function of Mφ, Tregs, Bregs and neutrophils can also be modulated, and have synergy in pathophysiological mechanisms of IMIDs.

## 5. Advanced Experimental and Clinical Studies of EVs in Inflammatory Diseases

### 5.1. Evidence from Different IMID Models

In psoriasis, EVs can target inflamed skin via both the gut–skin axis and local skin administration and provide promising therapeutic effect, reducing epidermal hyperplasia and alleviating both skin and systemic inflammation [66]. Moreover, because of immunomodulation, differentiation and regeneration of MSCs and their derivatives, they become potential therapeutic agents for chronic skin inflammatory diseases, including atopic dermatitis (AD) and psoriasis [67]. Scientists also explored whether skin inflammation in vivo can be controlled by the local delivery of curcumin–albumin-EVs (CA-EVs) applying dissolvable microneedle arrays (dMNAs), proving that they can block and reverse in vivo skin inflammation in mouse and rat models effectively [68].

EVs are not only significant in the progression of ALI/ARDS, but also play a crucial role in the treatment. In sepsis-related ARDS, the most fatal type of ARDS, circulating EVs was proven to increase and aggravate organ injury by promoting pro-inflammatory M1φ polarization of monocytes [69]. This influence can be mitigated by reducing vascular cell adhesion molecule 1 (VCAM1) levels in EVs or blocking integrin subunit alpha 4 (ITGA4) on monocytes. In addition, MSC-EVs can exert beneficial therapeutic effects in H1N1 influenza virus and coronavirus 2 (SARS-CoV-2)-infected mice models, respectively, improving survival and reduced signs of lung damage in mice [70]. Another research on chronic obstructive pulmonary disease (COPD) indicated that suppression of cytokine signaling 3 (SOCS3) expression and its regulation is in relation to the inflammation [71]. The SOCS3 level of Mφ-EVs in bronchoalveolar lavage (BAL) may help assess the grade of inflammation and possible progression of COPD. Meanwhile, miRNAs could downregulate SOCS3 in smokers, leading to a higher risk of incidence of COPD.

In inflammation-driven arthritis, the amount of EVs in serum increases and they express more of the surficial IL6 signal transducer [72]. Researchers designed engineered EVs with signaling-incompetent decoy receptors, capturing excessive IL6 and blocking IL6 trans-signaling in vitro, showing great anti-inflammatory possibility. Additionally, to identify the advantages of cell-free therapy, a group of scientists used MSC-miR-21(−) and MSC-miR-21(−)-EVs to treat osteoarthritis (OA) [19]. MSC-miR-21(−)-EVs exhibited superiority on reducing serum cytokines and chemokines in treated animals, in addition to increasing the senescence-associated secretory phenotype (SASP) and inflammatory markers, indicating the potential of non-cell therapies.

### 5.2. Clinical Transformation Progress

In order to further recruit EVs to the targeted area, a surface modification approach of EVs based on the combination of bioorthogonal copper-free click chemistry (BCC) and metabolic glycoengineering (MGE) was proposed [73]. Therefore, PEGylated hyaluronic acid markers that specifically bind to CD44-expressing cells are representative targeting moieties on the EVs’ surface. In addition, reprogramming of natural EVs through genetic engineering and other approaches offers the tantalizing prospect of expanding the therapeutic capabilities of EVs beyond their native functions and properties [74]. Moreover, administration is important for the expansion of the utilization of EVs as well. Thus, due to the multiple advantages of oral drug delivery, there is growing interest in developing advanced EVs for oral delivery of different therapeutic agents [75]. Orally administered EVs (O-EVs) enhance drug delivery by encapsulating therapeutic agents, ensuring targeted release and reducing toxicity, and exhibit excellent biocompatibility and stability, providing a new approach for anti-inflammatory therapy [76].

## 6. Challenges and Future Prospects

The standardized production, purification and characterization of EVs, which are considered as the limitations and challenges of large-scale application, remain technical difficulties that need to be faced in the future. Recent research showed that high-performance anion exchange chromatography purification of EVs enhances purity and anti-inflammatory efficacy [77]. Acoustic trapping was also applied to isolate and enrich EVs from their origins [78]. In addition, scientists have proposed an immuno-affinity methodology to further separate the inflammation-associated EVs subpopulations [79]. How to track EVs in vivo and their pharmacokinetics still need to be studied in-depth as well. We believe that once these difficulties are resolved, the advantages of MSC-EVs, including self-replication inability, lower risk of immune rejection, ectopic differentiation, genetic stability and tumor formation will be magnified.

As for the future prospects, two very important scientific questions urgently need to be answered. One is the verification of the causal relationship between specific molecular load and function, and the other is the heterogeneity of Mφ subsets (e.g., tissue-resident vs. monocyte-derived) in response to EVs. Clinically, researchers should work on continuously developing EVs-based cell-free therapies. In addition, combined therapy is also a promising research direction, for example, EVs with biomaterial scaffolds to enhance local delivery. We sincerely hope that our work will provide more references for related research in the future.

## Data Availability

The original contributions presented in the study are included in the article. Further inquiries should be directed at the corresponding authors.

## Abbreviations

The following abbreviations are used in this manuscript:

SYSU	Sun Yat-Sen University
EVs	Extracellular vesicles (EVs)
MSCs	Mesenchymal stem cells
Mφ	Macrophages
MSC/Mφ axis	Mesenchymal stem cells/macrophages axis
RA	Rheumatoid arthritis
IBD	Inflammatory bowel disease
M1φ	M1-like macrophages
M2φ	M2-like macrophages
MSC-EVs	Mesenchymal stem cell-derived EVs
Mφ-EVs	Macrophage-derived EVs
UC-MSCs	Umbilical cord mesenchymal stem cells
AMSCs	Amniotic mesenchymal stem cells
BM-MSCs	Bone marrow mesenchymal stem cells
AD-MSCs	Adipose tissue mesenchymal stem cells
TLRs	Toll-like receptors
PAMPs	Pathogen-associated molecular patterns
DAMPs	Damage-associated molecular patterns
LPS	Lipopolysaccharide
HMGB1	High-mobility group box 1 protein
NF-κB	Nuclear factor kappa-light-chain-enhancer of activated B cells
Bregs	Regulatory B cells
IRF5	Interferon regulatory factor 5
STAT1	Signal transducer and activator of transcription 1
iNOS	Inducible nitric oxide synthase
TNF-α	Tumor necrosis factor α
IL6	Interleukin 6
CD86	Cluster of Differentiation 86
IRF4	Interferon regulatory factor 4
STAT6	Signal transducer and activator of transcription 6
IL10	Interleukin 10
CD206	Cluster of Differentiation 206
DMARDs	Disease-modifying antirheumatic drugs
IMIDs	Immune-mediated inflammatory diseases
TGF-β	Transforming growth factor β
CD200	Cluster of Differentiation 200
CD200R	Cluster of Differentiation 200 receptors
HIF-1α	Hypoxia-inducible factor 1α
miRNAs	Micro-RNAs
miR-223	MicroRNA-223
PKNOX1	PBX/Knotted 1 Homeobox 1
miR-181c	MicroRNA-181c
TLR4	Toll-like receptor 4
miR-21	MicroRNA-21
PTEN	Phosphatase and tensin homolog
Akt	Protein kinase B
BBB	Blood–brain barrier
MVs	Microvesicles
ApoVs	Apoptotic vesicles
lncRNA	Long non-coding RNA
CD9	Cluster of Differentiation 9
CD81	Cluster of Differentiation 81
FasL	Fas ligand
MHC	Major histocompatibility complex
Tregs	Regulatory T cells
miR-155-3p	MicroRNA-155-3p
miR-148a-3p	MicroRNA-148a-3p
miR-1a-3p	MicroRNA-1a-3p
miR-143-3p	MicroRNA-143-3p
STAT3	Signal transducer and activator of transcription 3
JNK	c-Jun N-terminal kinase
miR-709	MicroRNA-709
M2φ-EVs	M2-like macrophages-derived EVs
ALI	Acute lung injury
ARDS	Acute respiratory distress syndrome
IL-1β	Interleukin 1β
NLRP3	NLR family pyrin domain-containing 3
PD	Periodontitis
Pg	Porphyromonas gingivalis
miR-143-3p	MicroRNA-143-3p
IGFBP5	Insulin-like growth factor-binding protein 5
miR-92a-3p	MicroRNA-92a-3p
SOST	Sclerostin
miR-625-3p	MicroRNA-625-3p
miR-671-5p	MicroRNA-671-5p
AAK1	Adaptor-associated protein kinase 1
TSG6	Tumor necrosis factor-inducible gene 6 protein
TLR2	Toll-like receptor 2
CD44	Cluster of Differentiation 44
IFN-γ	Interferon gamma
JAK	Janus kinase
COX-2	Cyclooxygenase-2
cAMP	Cyclic adenosine monophosphate
PKA	Protein kinase A
IL12	Interleukin 12
LDHA	Lactate dehydrogenase
PKM2	Pyruvate kinase M2
MyD88	Myeloid differentiation primary response 88
miR-146a	MicroRNA-146a
UTR	Untranslated region
mRNA	Messenger RNA
IL-1R	Interleukin-1 receptor
IRAK	IL-1R-associated kinase
IκBα	Nuclear factor of kappa light polypeptide gene enhancer in B-cells inhibitor alpha
SOD2	Superoxide dismutase 2
ROS	Reactive oxygen species
IKK	IκB kinase
PI3K	Phosphatidylinositol 3-kinase (PI3K)
PIP3	Phosphatidylinositol-3,4,5-triphosphate
mTOR	Mammalian target of rapamycin
mTORC1	mTOR complex 1
TSC1	Tuberous sclerosis complex 1
TSC2	Tuberous sclerosis complex 2
FAO	Fatty acid oxidation
PGC-1α	Peroxisome proliferator-activated receptor gamma coactivator 1-alpha
OXPHOS	Oxidative phosphorylation
IL4	Interleukin 4
IL13	Interleukin 13
JAK1	Janus kinase 1
JAK3	Janus kinase 3
IL-4Rα	Interleukin 4 receptor α
SOCS	Suppressor of cytokine signaling
PPARγ	Peroxisome proliferator-activated receptor γ
IL18	Interleukin 18
AD	Atopic dermatitis
CA-EVs	Curcumin–albumin-EVs
dMNAs	Dissolvable microneedle arrays
VCAM1	Vascular cell adhesion molecule 1
ITGA4	Integrin subunit alpha 4
SARS-CoV-2	Coronavirus 2
COPD	Chronic obstructive pulmonary disease
SOCS3	Suppressor of cytokine signaling-3
BAL	Bronchoalveolar lavage
OA	Osteoarthritis
SASP	Senescence-associated secretory phenotype
BCC	Bioorthogonal copper-free click chemistry
MGE	Metabolic glycoengineering
O-EVs	Orally administered EVs
